# Correlation between Changes in Local Earth’s Magnetic Field and Cases of Acute Myocardial Infarction

**DOI:** 10.3390/ijerph15030399

**Published:** 2018-02-26

**Authors:** Gediminas Jaruševičius, Tautvydas Rugelis, Rollin McCraty, Mantas Landauskas, Kristina Berškienė, Alfonsas Vainoras

**Affiliations:** 1 Department of Cardiology, Hospital of Lithuanian University of Health Sciences Kauno klinikos, Kaunas 50161, Lithuania; gedijaru@yahoo.com; 2 Cardiology Institute, Lithuanian University of Health Sciences, Kaunas 44307, Lithuania; 3 Academy of Medicine, Lithuanian University of Health Sciences, Kaunas 44307, Lithuania; trugelis@gmail.com; 4 HeartMath Institute, Boulder Creek, CA 95006, USA; rollin@heartmath.org; 5 Department of Mathematical Modelling, Kaunas University of Technology, Kaunas 51368, Lithuania; mantas.landauskas@ktu.lt; 6 Sport Institute, Lithuanian University of Health Sciences, Kaunas 47181, Lithuania; k.berskiene@gmail.com

**Keywords:** geomagnetic field, Earth’s magnetic field, myocardial infarction, magnetic field, cardiology, acute coronary syndrome

## Abstract

The impact of changes in the geomagnetic field on the human body remains the subject of studies across the world, yet there is no consensus. Current studies are observing effects that require further work by researchers in order to find out the mechanisms that would allow a proper assessment of the correlations between the Earth‘s magnetic field variations and changes in human organisms. The main purpose of this study was to investigate possible correlations between the strength of time-varying aspects of the local Earth’s magnetic field and incidence of myocardial infarctions. Study participants included 435 males and 268 females who had diagnosis of myocardial infarction during the period of 1 January 2016 to 31 December 2016 and attended the Department of Cardiology at the Hospital of Lithuanian University of Health Sciences (LUHS), Kauno klinikos. Time varying magnetic field data was collected at the magnetometer site located in Lithuania. After mathematical analysis, the results support the hypothesis that the Earth’s magnetic field has a relationship between the number of acute myocardial infarction with ST segment elevation (STEMI) cases per week and the average weekly geomagnetic field strength in different frequency ranges. Correlations varied in different age groups as well as in males and females, which may indicate diverse organism sensitivity to the Earth’s magnetic field.

## 1. Introduction

### 1.1. Cell Regulation

The hypothesis that the time varying magnetic fields of the earth may be regulatory mechanism of cells acting through the ion cyclotron resonance mechanism has been proposed [1]. It is claimed that every living organism has specific sensitivity to the strength and frequency of fluctuations of magnetic fields [1,2]. Laboratory findings demonstrate the effect of ion cyclotron mechanism on extracted myocardial cell regulation [3]. However, according to recent publications, it remains unclear whether the local magnetic field of the Earth has an effect on the regulation of human heart cells. We started investigating this at our university in 2014, when we signed agreement of cooperation between Lithuanian University of Health Sciences (LUHS) and Heartmath Institute, located in California, USA. Thanks to the directorate of the institute, Lithuania received and began to operate extremely sensitive magnetometer (pT sensitivity), the only one of a kind in Europe. Currently, there are six such magnetometers across the globe: USA, Canada, Saudi Arabia, New Zealand, South Africa, and Lithuania. By using the magnetometer’s live data we can observe changes in the local earth’s time varying magnetic fields in Lithuania and compare it with medical data.

### 1.2. Regulation of Cardiovascular System 

Scientists have investigated the effect on cardiovascular system of healthy humans in the absence of earth‘s magnetic field. When healthy subjects were isolated from the magnetic field and compared to a control group, a significant increase of 17% in capillary blood flow and average reduction of 2 mmHg in diastolic blood pressure on was found [4].

In other studies, researchers evaluated how changes in the geomagnetic field affects cardiovascular regulation under laboratory conditions. Baroreflex sensitivity, estimated from blood pressure and heart rate responses to intravenous injections of phenylephrine and nitroprusside, showed significant negative correlations between increasing geomagnetic field disturbance and baroreflex sensitivity, heart rate variability, and arterial blood pressure. These findings support the theory that geomagnetic field disturbances affect neural cardiovascular regulatory centers [5]. The decrease of baroreflex sensitivity may lead to higher mortality after myocardial infarction [6]. 

**Figure 1:**
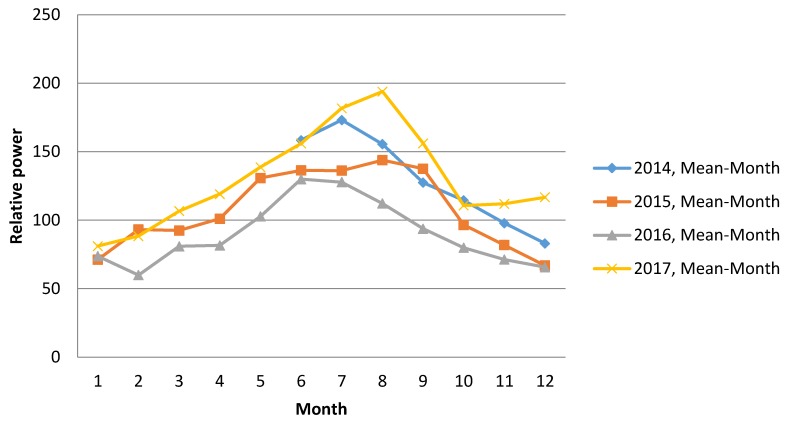
The changes of power of local Earth’s magnetic field in Lithuania (GCI003) during 2014–2017 years.

**Figure 2:**
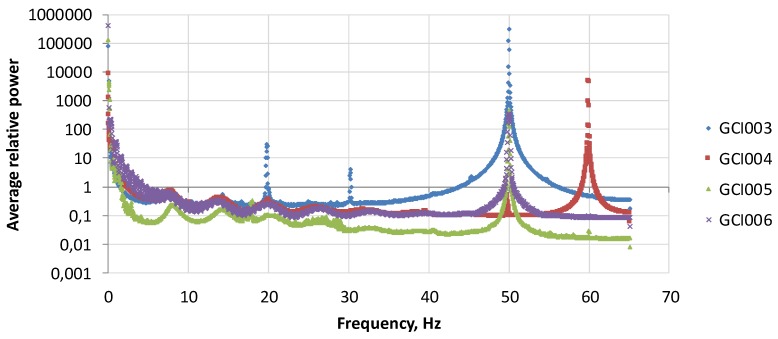
Schumann resonances 24 April 2016 through 30 April 2016 for magnetometers GCI003—Lithuania; GCI004—Canada; GCI005—New Zealand; and GCI006—South Africa.

The Earth‘s magnetic field is a constantly changing. It changes daily, throughout the week, month, and year. During winter spectral power of the local magnetic field of the Earth decreases (Figure 1), begins to increase in spring and reaches the peak in summer. In autumn, the strength of magnetic field starts to decrease to the lowest point in winter (Lithuania GCI003 magnetometer data from https://www.heartmath.org/research/global-coherence/gcms-live-data/). These changes may affect the processes occurring in human organisms. In the literature on health disturbances, it has been found that both weak and strong magnetic fields are associated with negative health outcomes [7,8]. We believe that different people may have different sensitivity to different frequencies of magnetic fields, but there may be potential differences due to age, gender, and health status. Low frequency magnetic fields are believed to have positive effects on humans, however, high frequencies may cause stress reactions to human regulatory systems [7,9]. To this purpose, we investigated correlations between the changes in geomagnetic fields in different frequency ranges and hospital admissions. The Schumann resonances at different locations over a six-day period are shown in Figure 2 [10].

In the estimated power curve on the frequency range, from 0 to 65 Hz there is a series of dominant Schumann resonance frequencies, which are divided into ranges that overlap with the EEG wave classification (as related processes): 0 to 3.5 Hz—Delta waves (P1); 3.5 to 7 Hz—Theta waves (P2); 7 to 15 Hz—Alpha waves (P3); 15 to 32 Hz—Beta waves (P4); 32 to 100 Hz—Gamma waves (P5). The latter high frequency waves are believed to cause stress effects on living organisms [11,12]. As stated in [12], “It is certain that gamma, like other brain rhythms, can provide a signature of cognitive state, as well as network dysfunction”.

### 1.3. Geomagnetic Field and Melatonin 

Studies have shown that changes in the time varying magnetic field above 80 Nt, over three hours significantly reduces melatonin levels in the body [13,14]. Indications for the use of melatonin for treatment are not approved in cardiology, although there are many researchers who suggest that melatonin may have a positive effect on people with ischemic heart disease [15]. In addition, antiarrhythmic action of melatonin during reperfusion in acute coronary syndromes has shown promise in a study [16]. Also, under laboratory conditions, it has been found that melatonin improves myocardial microcirculation [17]. Thus, reduced levels of melatonin, along with other individual factors, may play a role in development of myocardial ischemia due to changes in geomagnetic field.

## 2. Materials and Methods 

### 2.1. Participants

**Figure 3:**
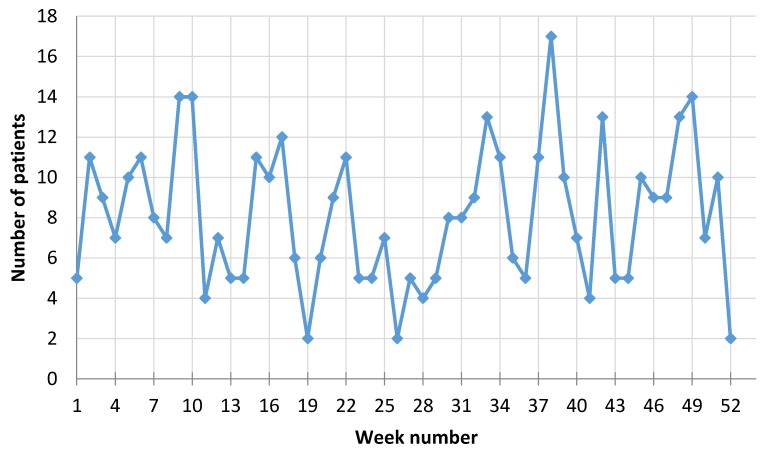
Number of men admitted with MI each week during 2016.

**Figure 4:**
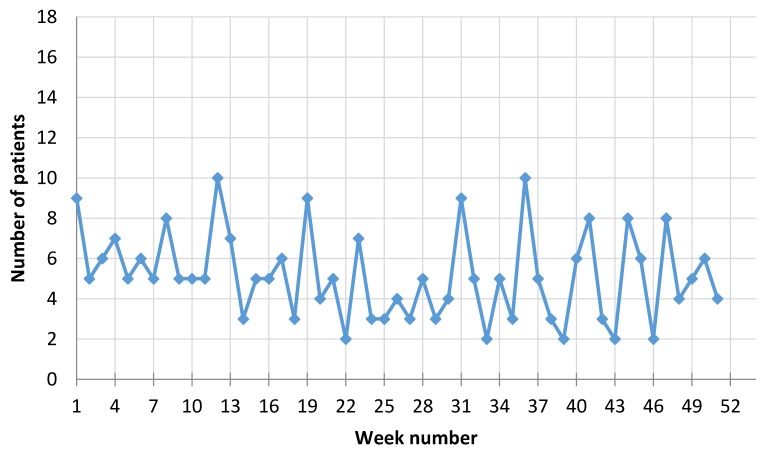
Number of women admitted with MI in each week during 2016.

There was a total of 703 participants in the study (435 men and 268 women) with a diagnosis of new myocardial infarction (MI) event during the period from 1 January 2016 through 31 December 2016, who attended the Cardiology Department of University Hospital at Lithuanian University of Health Sciences (BEC-MF-254). Mean age of the men was 63.44 ± 11.65, (Me = 63) and 73.21 ± 10.45 (Me = 75) for women. Men group we have divided into two groups according Me—younger group with age <63 years. (N = 229, mean age ± Stdev = 54.57 ± 3.43), and older group age >63 years. (N = 206, mean age ± Stdev = 73.53 ± 4.84). The men’s mean age was almost 10 years younger than the women’s group, but older men group was just the same as women group age (no significant differences were observed). All the patients were admitted to the Cardiology Department with diagnosis of MI. All the patients were highly symptomatic during the time of arriving to the hospital. Figure 3 shows the number of male patients admitted for MI during each week in 2016 and Figure 4 for females. 

In our study, we divided 2016 year into two time intervals, first half of the year and second half of the year: 1 (weeks 1 through 26) and 2 (weeks 26 through 52). In a more detailed analysis we further divided the male group into two age groups (as mentioned earlier): younger than 63 and older than 63. We did not divide the female patient group due to the smaller number of females.

### 2.2. Magnetometer Data 

The local time varying magnetic field intensity was measured using a local magnetometer located in Lithuania (Coordinates: Latitude: 55.634068 Longitude: 23.704563), which is part of the Global Coherence Monitoring Network. Two magnetic field detectors (Zonge Engineering Inc., Tucson, AZ, USA) ANT4 are positioned in north/south and east/west orientation. Data used in the analysis is from the east–west direction. Signals from the magnetometers were digitized with a 24-bit data acquisition system (Symmetric Research, Las Vegas, NV, USA) at a rate of 130 Hz and uploaded hourly to a cloud data storage site for offline processing. The overview of the magnetometer’s data is available on web page [18]. Hourly data files were downloaded to a personal computer (PC) workstation for post processing where were each hourly data file was transformed into consecutive 30 s long segments. The power spectral density (PSD) was calculated for each segment. All PSD segments for each hour were then averaged together. The sum of the PSD in the frequency range from 0–66 Hz was calculated for each hour in the study period. Mean power of local magnetic field fluctuations in Lithuania, measured in pT^2^ in five different frequency ranges where overlaps between the Schumann resonance and EEG frequency ranges (we named them as SDelta (0; 3.5 Hz), STheta (3.5; 7 Hz), SAlpha (7; 15 Hz), SBeta (15; 32 Hz), and SGamma (32; 66 Hz) to distinguish them from the EEG bands). 

### 2.3. Spectral Analysis of the Magnetometer Data

Consider magnetic field intensity ${\left\{I_{t}\right\}}_{t=0}^{N-1}$, where t is discrete time variable.

(1) $$f(\omega )=\sum_{t=0}^{N-1}I_{t}\cdot e^{\frac{-2\pi it\omega }{N}},t\in Z.$$

In order to transform ${\left\{I_{t}\right\}}_{t=0}^{N-1}$ to the frequency domain the discrete Fourier transform (DFT) (Equation (1)) was used [19]. The drawback of DFT is that one cannot observe the change in spectral density over time unless sequentially computing DFT. To achieve this the discrete time short time Fourier transform (STFT) was employed.

(2) $$F(\tau ,\omega )=\sum_{t=-\infty }^{\infty }I_{t}\cdot \xi (t-\tau )e^{-it\omega },t\in Z.$$

STFT for ${\left\{I_{t}\right\}}_{t=0}^{N-1}$ is represented by Equation (2). In fact this is essentially the analogue for Equation (1) but applied to the function $I_{t}\cdot \xi (t-\tau )$. ξ(t) is a so called windowing function which has a value close to 1 in a subdomain of t centered on 0 and a value close to 0 elsewhere. The units of f(ω) and F(τ,ω) are pT·s due to the fact that the intensity of the magnetic field is measured in pT.

(3) $$S(\tau ,\omega )={\left|F(\tau ,\omega )\right|}^{2}.$$

Spectrograms investigated in this work is the squared modulus of STFT (Equation (3)). Originally units of a spectrogram would be ${\mathrm{pT}}^{2}\cdot s^{2}$. S(τ,ω) is often referenced as power spectral density. Thus the value of S(τ,ω) is interpreted as signal power at the time interval Δτ and at the frequency range Δω. 

More detailed algebraic and spectral analysis of local magnetic field intensity is presented in article [20] and Schumann resonances calculated from magnetometers data are shown in Figure 2.

### 2.4. Statistical Analysis

Nonparametric Mann–Whitney U test for the comparison of two independent samples was used. Pearson correlation coefficient for the linear correlation between two variables was calculated. The level of *p* < 0.05 was considered statistically significant.

## 3. Results

**Figure 5:**
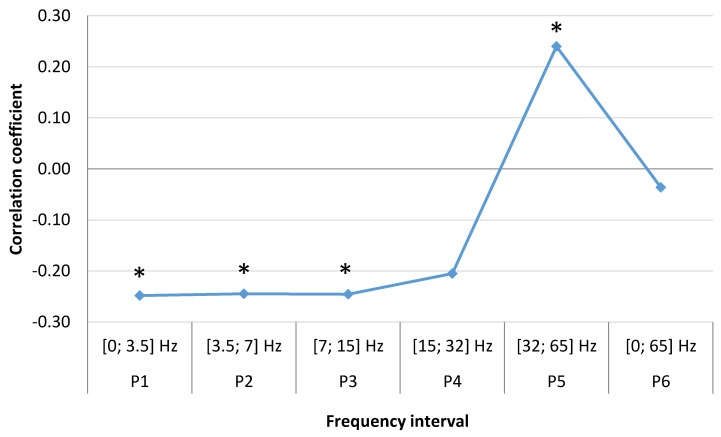
Correlation between number of weekly MI cases in women and mean magnetic power in different frequency ranges spanning a time of one week (data from LUHS, Cardiology clinic, 2016), * *p* < 0.05.

We found a significant relationship between number of acute myocardial infarction with ST segment elevation (STEMI) cases per week and the average weekly geomagnetic field strength in different frequency ranges. In the female group (N = 268), we found single positive correlation coefficient P5 (SGamma) [32; 65] Hz, (r = 0.25, *p* = 0.037), which indicates that higher magnetic field intensity in this frequency range is significantly associated with increased number of STEMI cases. In other low frequency ranges, we observed negative correlation coefficients: P1 (SDelta) [0; 3.5] Hz, (r = −0.24, *p* = 0.043), P2 (STheta) [3.5; 7] Hz, (r = −0.24, *p* = 0.043), P3 (SAlpha) [7; 15] Hz, (r = −0.24, *p* = 0.043), P4 (SBeta) [15; 32] Hz, (r = −0.20, *p* = 0.078), P6 [0; 65] Hz, (r = −0.04, *p* = 0.389) (Figure 5). Negative correlations are associated with decrease of STEMI cases, thus P6 correlation, including all intervals, did not demonstrate significance.

**Figure 6:**
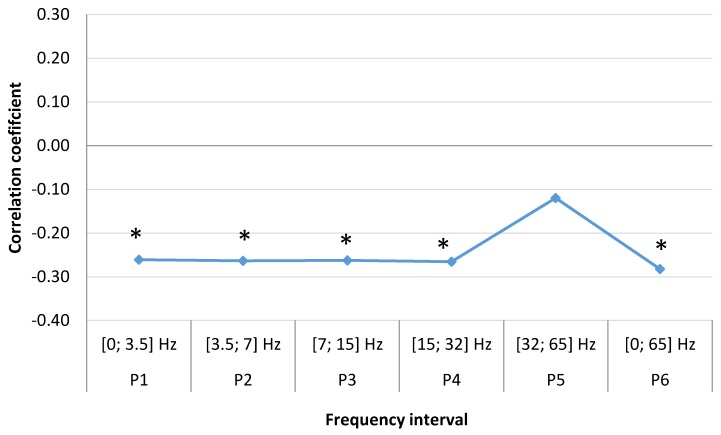
Correlation between men’s weekly MI number and mean magnetic power in different frequency ranges spanning a time of one week (data from LUHS, Cardiology clinic, 2016), * *p* < 0.05.

In the male group (N = 435), the weekly correlation coefficients (Figure 6) is similar to the female group, however, all coefficients are negative: P1 (SDelta) [0; 3.5] Hz, (r = −0.26, *p* = 0.031), P2 (STheta) [3.5; 7] Hz, (r = −0.26, *p* = 0.031), P3 (SAlpha) [7; 15] Hz, (r = −0.26, *p* = 0.031), P4 (SBeta)[15; 32] Hz, (r = −0.27, *p* = 0.026), P5 (SGamma) [32; 65] Hz, (r = −0.12, *p* = 0.198), P6 [0; 65] Hz, (r = −0.28, *p* = 0.022). Unlike females, we observe non-significant changes in SGamma range, which may indicate slightly different sensitivity of different sexes to the changes of the Earth‘s magnetic field. It should be considered that all these frequencies are present at the same time, therefore, the negative SGamma frequency range effect may not outweigh the positive effects of other frequency ranges (SDelta, STheta, SAlpha, SBeta).

**Figure 7:**
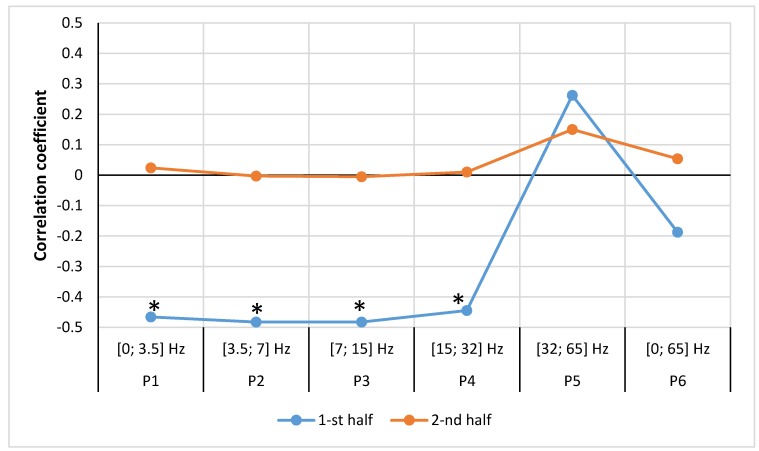
Correlation between number of women weekly cases of MI and mean magnetic power in different frequency ranges spanning a time of one week (data from LUHS, Cardiology clinic, 2016), * *p* < 0.05.

After dividing our study period into two halves (weeks 1–26 and weeks 27–52), we found that in the female group, the relationship between the number of STEMI cases and geomagnetic field strength was different between the two time periods. In the first half of the year, only the P5 [32; 65 Hz] range was positively correlated, although it was not significant, (r = 0.25, *p* = 0.109). In other frequency ranges stronger and significant negative correlations were observed: P1 (SDelta) [0; 3.5] Hz, (r = −0.47, *p* = 0.008), P2 (STheta) [3.5; 7] Hz, (r = −0.48, *p* = 0.007), P3 (SAlpha) [7; 15] Hz, (r = −0.48, *p* = 0.007), P4 (SBeta) [15; 32] Hz, (r = −0.45, *p* = 0.011), P6 [0; 65] Hz, (r = −0.19, *p* = 0.176). In the second half of the year, P1–P4 ranges demonstrated non-significant, close to zero correlations, P5 (SGamma) [32; 65] Hz, (r = 0.17, *p* = 0.203). These results may suggest, that the sensitivity of females to the fluctuations of geomagnetic field’s power is changing throughout the year and the strong to moderate correlation disappears in the second half of the year (Figure 7).

**Figure 8:**
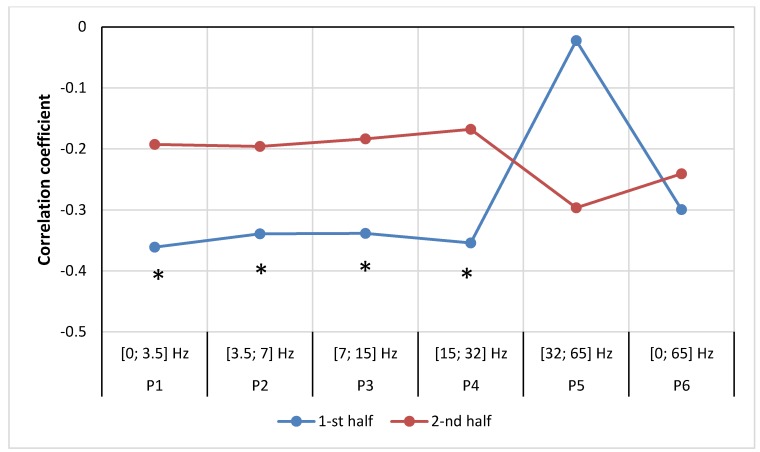
Correlation between number of men weekly cases of MI and mean magnetic power in different frequencies for first and second half of year 2016 (data from LUHS, Cardiology clinic, 2016 year), * *p* < 0.05.

After dividing the male group into the same two periods, different correlations between the number of STEMI cases and the geomagnetic field strength in first and second periods were observed. In first half of the year, non-significant correlations were observed in the frequency ranges P5 (SGamma) [32; 65] Hz, (r = −0.02, *p* = 0.461) and P6 [0; 65] Hz, (r = −0.30, *p* = 0.068), however other ranges revealed significant negative correlations: P1 (SDelta) [0; 3.5] Hz, (r = −0.36, *p* = 0.035), P2 (STheta) [3.5; 7] Hz, (r = −0.34, *p* = 0.045), P3 (SAlpha) [7; 15] Hz, (r = −0.36, *p* = 0.035), P4 (SBetta) [15; 32] Hz, (r = −0.35, *p* = 0.040). In the second half of the year, all correlations were non-significant (Figure 8).

In addition, the male group was divided into two groups according to age, 63 and below and 64 and above. In both groups P5 (SGamma) [32; 65] Hz range correlations were weaker than in the other frequency ranges. However, there was no significant difference between both group correlations in all ranges (U = 10; z = −1.28, *p* = 0.241). Despite that, a tendency of stronger correlations in the older males group was observed.

**Figure 9:**
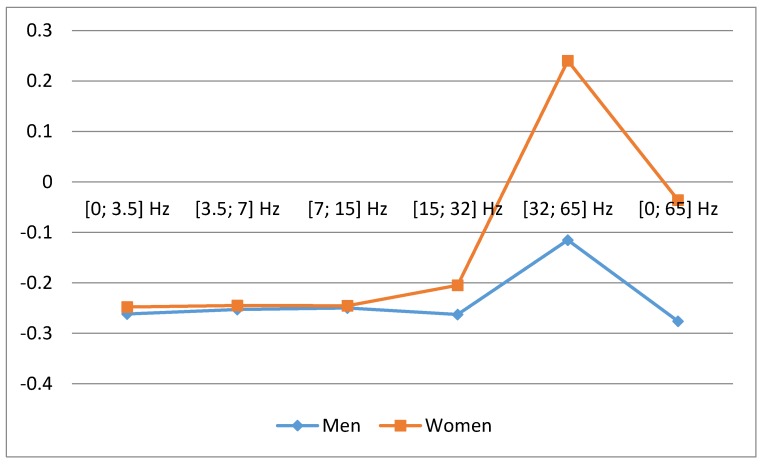
Correlation coefficients between number of men and women weekly cases of MI and mean magnetic power in different frequencies for older (>63 years) men group and women group for all year 2016. Difference between genders is non-significant.

To compare and discuss differences in correlations between all men and all women is difficult because in our country we have even 10 years’ difference between our male and female patients’ age we cannot say the differences are caused by gender or difference in mean age (Figure 5 and Figure 6). We have taken the older men’s group and compared year correlations with the women’s group—in both those groups, there was no difference in age. In Figure 9, correlations are presented in different frequency ranges for older men and women groups. Here we see, that differences between genders appear only in higher frequencies—SBetta and SGamma (non-significant), but in lower frequencies, we see just the same correlations for both genders. For the older patient reactions, men and women, to Earth’s magnetic field (EMF) different frequency bands in year interval are similar. 

**Figure 10:**
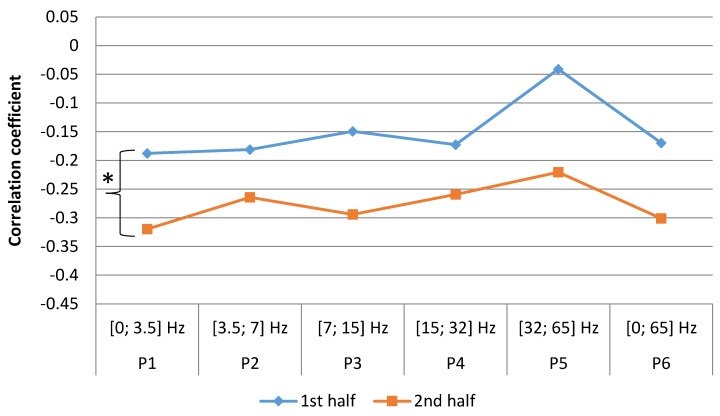
Correlation between number of men weekly cases of MI and mean magnetic power in different frequencies for younger (<63 years) men group for first and second half for year 2016 (data from LUHS, Cardiology clinic, 2016), * *p* < 0.05.

Based on correlation trend (Figure 10), we could see that similar ratio between the number STEMI cases and <63 y.o. males correlations was maintained in all magnetic field frequency ranges. Differences between both halves of year were significant (U = 0; z = −2.882; *p* = 0.002).

**Figure 11:**
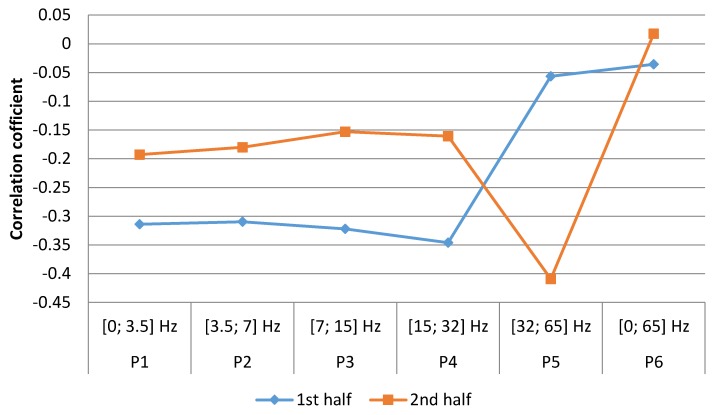
Correlation between number of men cases of MI per week and mean magnetic power in different frequencies for older (>63 years age) men group for first and second half for year 2016 (data from LUHS, Cardiology clinic, 2016).

In the older-than-63 y.o.-male group (Figure 11), a different correlation tendency was observed in comparison to younger-than-63 y.o. group (Figure 10). However, there was no significant difference (U = 14; z = −0.641; *p* = 0.589) when comparing the correlations between the two halves of the year in all frequency ranges. [0; 3.5], [3.5; 7], [7; 15], [15; 32] (Hz) ranges had stronger correlation tendency in weeks 27–52. The opposite result was obtained in weeks 1–26 range P5 (SGamma) [32; 65] Hz, (r = −0.4, *p* = 0.021), weeks 27–52 (r = −0.05, *p* = 0.404). In the first half of the year significant negative correlation was observed in frequency range P4 (SBetta) [15; 32] Hz, (r = −0.346, *p* = 0.042).

## 4. Study Limitations

This study has several limitations. First, this study was performed about elderly hospitalized patients, making it difficult to apply the results to general population of all ages. Secondly, we did not evaluate solar activity or other weather conditions that may have additional effect besides changes in local geomagnetic field. An additional limitation of this study is that we did not evaluate the presence of other diseases or conditions which could also be affected by changes in geomagnetic field strength.

## 5. Discussion

The data we obtained by analyzing different sexes and different age group correlations with geomagnetic field strength revealed unequal relationships between these processes. Obviously, without direct influence of the field, we could not imagine the dynamics and diversity of correlations. In the first half of the year, we observed higher sensitivity to geomagnetic field of number of female MIs, which could not be explained by classical physiology. Although, we begin to understand the principal effects of the field to the living organism with circadian rhythms (The Nobel Prize in Physiology or Medicine 2017 for Jeffrey C. Hall, Michael Rosbash and Michael W. Young “for their discoveries of molecular mechanisms controlling the circadian rhythm”). Nevertheless, it is still unknown what modulates these changes in longer periods of time. The strength of our work is that we did analysis of different geomagnetic field frequencies and results revealed that different frequency ranges have different correlations with presence of myocardial infarctions. Lithuanian researchers in another study have found correlation between heart rate variability and geomagnetic field strength, their study has shown that autonomic nervous system responds to solar and geomagnetic activity [21,22,23]. Moreover, in another study which included patients from Lithuania, researchers found out that number of acute myocardial infarction events after low geomagnetic field activity and high cosmic rays days increased by a fifth, their obtained results indicate that geomagnetic field may be related to development of myocardial infarction [24,25]. Our study results indicate that the effects of changes in local geomagnetic field should be studied analyzing field’s strength in frequency ranges. Ability to measure local changes of low frequency geomagnetic field provides an opportunity for researchers to begin more detailed future research.

## 6. Conclusions

1. Both male and female cases of MI are related to seasonal changes of local geomagnetic field.
2. In the first and second half of the year, correlations vary in all studied patient groups.
3. In the younger males group and in the elderly males group, correlations varied between cases of MI and geomagnetic field activity.
4. Lower number of myocardial infarction cases has negative correlation with the changes in local geomagnetic field (SDelta, STheta, SAlpha, SBeta frequency ranges).
5. The changes in the local geomagnetic field high frequency (SGamma) range are correlated with a higher number of myocardial infarction cases.
